# Surface engineering of chak’o nano-clay with iron oxide and APTES for enhanced heavy metal adsorption in water treatment

**DOI:** 10.12688/f1000research.162878.1

**Published:** 2025-03-27

**Authors:** Russbelt Yaulilahua-Huacho, Liliana Asunción Sumarriva-Bustinza, Jorge Luis Huere-Peña, Carlos Dueñas-Jurado, Edwin Javier Ccente-Chancha, José Carlos Ayuque-Rojas, Cesar Castañeda-Campos, Miriam Liz Palacios-Mucha, Rubén Garcia-Ticllacuri, Herbert Rodas-Ccopa, Zaida Olinda Pumacayo-Sanchez, Manuel Castrejón-Valdez, María Clarisa Tovar-Torres, Russell Mejia-Cayllahua, Bryan Jefferson Abollaneda-Altamirano, Indalecio Quispe-Rodríguez

**Affiliations:** 1 Facultad de Ciencias de Ingenieria, National University of Huancavelica, Avenida Agricultura 319-321, Paturpampa, Huancavelica, 09001, Perú, Huancavelica, Peru; 2 Facultad de Ciencias, Universidad Nacional de Educación Enrique Guzmán y Valle, Avenida Enrique Guzman y Valle Nº 951, La Cantuta, Chosica, Lurigancho, Lima, 150118, Perú, Lima, Peru; 3 Facultad de Ingeniería, Universidad Nacional José María Arguedas, Jirón Juan Francisco Ramos N° 380, Andahuaylas, 030201, Perú, Juan Francisco, Peru; 4 Facultad de Ingeniería, Universidad Peruana Los Andes, Avenida Av. Giráldez 230, Huancayo, 12002, Perú, Huancayo, Peru; 5 Facultad de Ingeniería de Minas Geología y Civil, Portal Independencia N° 57, Huamanga, Universidad Nacional de San Cristobal de Huamanga, Ayacucho, 050101, Perú, Ayacucho, Peru

**Keywords:** Chak’o nano-clay, surface modification, heavy metal removal, adsorption kinetics, Mantaro river, environmental remediation

## Abstract

**Background:**

The heavy metal contamination of water leads to major environmental and health problems throughout the Mantaro River basin area. The study assesses the adsorption capacity of surface-modified chak’o nano-clay for extracting lead (Pb ^2+^) and arsenic (As ^5+^).

**Methods:**

Surface modifications of unmodified nano-clay were achieved using through Aminopropyltriethoxysilane (APTES), iron oxide (Fe _3_O _4_), and combined APTES + Fe _3_O _4_ modifications of unmodified nano-clay particles. Adsorption performance was assessed through batch experiments over 8 hours. BET and XPS analyses were conducted to determine surface area, pore volume, and functional group availability. Adsorption kinetics were modeled using a pseudo-second-order model, and equilibrium data were analyzed using the Langmuir isotherm.

**Results:**

The dual surface modification produced maximum removal capacities which led to a 95–100% removal performance of Pb ^2+^ and As ^5+^ over 8 hours. The BET and XPS analysis demonstrated that surface area (300 m ^2^/g to 375 m ^2^/g) and pore volume (0.420 cm ^3^/g to 0.600 cm ^3^/g) as well as functional group availability increased substantially thus resulting in improved adsorption. The pseudo-second-order model fit well for adsorption kinetic data while equilibrium data fit Langmuir isotherm behavior to describe monolayer adsorption. The dual treatment of APTES in combination with iron oxide generated an adsorbent with better magnetic properties and electrical conductivity thus improving its recovery potential and structural stability. The dual-modified nano-clay showed high stability during three cycles through desorption tests because it maintained more than 90% of its original adsorption capacity throughout the process.

**Conclusion:**

The research shows that specialized surface treatments deliver advanced heavy metal absorption abilities to chak’o nano-clay which positions it as a leading choice for sustainable water system heavy metal remediation.

## Introduction

Environmental challenges stem from heavy metal pollution since these pollutants harm human health as well as ecosystem structures. Heavy pollutants enter the environment through several sources including manufacturing plants and mining sites and agricultural activities and trash disposal sites. The three major problematic heavy metals include lead (Pb ^2+^) along with arsenic (As ^5+^) and cadmium (Cd ^2+^). These heavy metals gather in water bodies and soils producing enduring ecological and health complications. The neurologic harm caused by Pb ^2+^ primarily affects children while As ^5+^ functions as a carcinogenic agent and Cd ^2+^ displays toxicity against bones and kidneys. Watersource contamination requires immediate heavy metal extraction because it protects environmental areas together with securing public health ( Kurhaluk et al., 2024). Scientists have adopted four main techniques to purify water from heavy metals: chemical precipitation followed by ion exchange and membrane filtration and finally adsorption. The adsorption method stands as an optimal and extensively researched water treatment technique because of its ease of application and low operational cost together with its ability to effectively eliminate different pollutants ( Rashid et al., 2021). Heavy metal ion recognition and surface stagnation proceeds with materials that draw and maintain these ions on their outer layers through physical and chemical surface forces such as van der Waals attraction and electrostatic binding and chemical attachment. The adsorption process depends on materials' capacity to adhere heavy metal ions by physical and chemical bond mechanisms which include van der Waals forces and electrostatic attraction and chemical bonding. Material effectiveness for adsorption purposes depends on parameters like surface area with pore size distribution as well as surface functionalization and material stability according to Mane et al. (2024). Nano clays and clay minerals increasingly interest scientists working in environmental remediation because they possess remarkable surface area properties alongside abundance and environmental compatibility and affordability. The adsorption properties of montmorillonite and bentonite nano-clays can easily be improved by modifying their surface hydroxyl groups. The surface modification of clays with different organic and inorganic elements enhances heavy metal adsorption through either added active sites or modified surface charges according to Zhang et al. (2021). APTES (3-aminopropyltriethoxysilane functions as a common organosilane modifier to create clay surfaces with added metal ion adsorption capabilities through its addition of -NH2 groups ( Mao and Gao, 2021).

The incorporation of iron oxide (Fe _3_O _4_) as a metal oxide modification leads to improved material stability and surface area while giving the material magnetic properties which enable simple post-adsorption separation ( Liu et al., 2020). Nano clays receive great benefit through the iron oxide nanoparticle addition because these materials show strong heavy metal adsorption based on their metal-chelating properties and highly reactive surfaces ( Emam, 2022). When organic APTES combine with inorganic iron oxide nanoparticles the materials gain parallel advantages which boost their absorption qualities and operational stability while extending their useful lifetime. This research examines the modification process of chak’o nano-clay by integrating APTES and iron oxide while studying its heavy metal removal aptitude. The main purpose of this study entails examining how chak'o nano-clay structural properties together with surface characteristics and Pb ^2+^ As ^5+^ Cd ^2+^ adsorption performance change following organic and inorganic modification procedures. The study examines performance factors in heavy metal remediation applications through evaluation of surface area, pore volume, surface functionality changes and adsorption isotherms and kinetic parameters of modified nano-clays. Multiple studies have already shown clay surfaces could successfully remove heavy metals from water yet more materials are needed with outstanding adsorption capacity and environmental stability and reusability capabilities ( Kalhori et al., 2013; Hacıosmanoğlu et al., 2022). The dual modification process applied in this research presents opportunities to generate an effective adsorbent which could facilitate large-scale water treatment operations. Research findings from this investigation support progress toward developing environmentally friendly and cost-efficient materials used to clean heavy metal pollution in water supplies.

## Methods

### Sampling sites

We selected research areas across the Mantaro watershed where contamination ranged from the minimal effects in Huancavelica (upper Mantaro Basin) and moderate pollution throughout middle Mantaro Basin sites Huancayo and Concepción, Jauja and extreme contamination near La Oroya due to industrial activities. Workers studied pollution dispersal by studying locations both in Junín Lake and the lower Mantaro Basin. Researchers measured pH and temperature as well as dissolved oxygen and electric conductivity and obtained double samples from water's surface and bottom layers of assessment sites. Half of the water samples were filtered for dissolved metals, but total metal analysis required unfiltered samples while preservation occurred at 4°C. The researchers used grab samplers to obtain sediment samples from the riverbed which they processed before preserving them for heavy metal evaluation. Experimental studies were conducted using polluted-site-derived iron oxide and APTES-modified chak’o nano-clay to determine its efficiency for heavy metal extraction such as lead, cadmium, and arsenic.

### Nano-clay preparation

The source of chak'o nano-clay originally came from natural deposits situated within the Mantaro River basin which scientists have studied for clay-based adsorption properties in the past. A succession of purification procedures involving sieve separation followed by sedimentation then centrifugation cleansed the raw nano-clay by extracting big particles and natural contaminants from the material. The purified clay received 105°C heat treatment for twenty four hours to evaporate all remaining moisture content. The X-ray diffraction (XRD) results confirmed the mineralogical nature of this material primarily included montmorillonite and kaolinite due to their reputational cation exchange capacity and strong adsorption behavior ( Gurav et al., 2024).

### Modification techniques

**APTES functionalization**

The APTES modification was employed to introduce amine (-NH _2_) functional groups, which enhance the adsorption of metal ions through electrostatic interactions and complexation ( Goharrizi et al., 2024). The process involved dispersing 10 g of purified chak’o nano-clay in 200 mL of anhydrous toluene, followed by the addition of 5% (v/v) APTES (Sigma-Aldrich, USA (Catalogue No. 440140)). The mixture was refluxed at 110°C under continuous stirring for 6 hours. After cooling, the modified clay was washed with ethanol and deionized water to remove excess reagents and then dried at 80°C for 12 hours. Fourier Transform Infrared Spectroscopy (FTIR) ( **Thermo Fisher Scientific Inc. USA)**) analysis confirmed successful functionalization through the appearance of characteristic N-H and Si-O-Si stretching vibrations at 3300 cm ^−1^ and 1080 cm ^−1^, respectively.

**Iron oxide coating**

Iron oxide (Fe _3_O _4_) ( **Sigma-Aldrich, USA (Catalogue No. 215422)**) modification was conducted via a co-precipitation method to enhance magnetic properties and facilitate recovery from aqueous solutions ( Khaled et al., 2024). In a typical procedure, 10 g of nano-clay was dispersed in 100 mL of deionized water and sonicated for 30 minutes. A solution containing Fe ^2+^ and Fe ^3+^ salts (molar ratio 1:2) was added, followed by the dropwise addition of 1 M NaOH ( **Merck KGaA, Germany (Catalogue No. 106462)**) until the pH reached 10. The mixture was continuously stirred at 80°C under nitrogen atmosphere to prevent oxidation. The resulting iron oxide-coated nano-clay was separated using a magnet, washed multiple times with deionized water, and dried at 60°C. X-ray photoelectron spectroscopy (XPS) (Kratos AXIS Supra+, Kratos Analytical Ltd., UK) confirmed the presence of Fe-O bonds, while magnetization studies indicated an increase in magnetic susceptibility.

**Dual modification (APTES + iron oxide)**

The combined modification process integrated both APTES functionalization and iron oxide coating to maximize adsorption efficiency. First, the nano-clay underwent APTES treatment following the previously described method. Subsequently, the APTES-modified clay was subjected to the iron oxide coating process. This dual modification aimed to enhance surface reactivity, improve stability across varying pH conditions, and enable easier separation using external magnets. Scanning Electron Microscopy (SEM) images revealed smoother, more uniform surfaces with increased porosity, and Brunauer-Emmett-Teller (BET) analysis indicated a significant rise in surface area, from 300 m ^2^/g (unmodified) to 375 m ^2^/g.

**Saturation magnetization (emu/g)**

A total of 1.0 g of each chak’o nano-clay variant (unmodified, APTES-modified, iron oxide-modified, and dual-modified) was prepared by drying in an oven at 60°C overnight to remove any moisture. The samples were placed in a vibrating sample magnetometer (VSM) (Lake Shore Cryotronics, Inc., USA) for measurement. Each sample was subjected to a magnetic field ranging from -15,000 Oe to +15,000 Oe at room temperature (25°C). The magnetization of each sample was recorded at regular intervals, and the saturation magnetization (Ms) was determined at the point where the magnetization curve reached its plateau.

**Electrical conductivity**

A suspension of 0.5 g of each Chak’o nano-clay variant (unmodified, APTES-modified, iron oxide-modified, and dual-modified) was prepared by dispersing the sample in 1 L of deionized water, using an ultrasonic bath for 30 minutes to ensure complete dispersion. Electrical conductivity was measured using a digital conductivity meter (Thermo Scientific Orion 5-Star, Thermo Fisher Scientific Inc., USA). The probe was immersed into the prepared suspension, and the conductivity was measured at room temperature (25°C) for each sample. Each measurement was performed in triplicate for consistency, and the conductivity was recorded in arbitrary units (AU).

**Stability**

Total of 1.0 g of each nano-clay variant (unmodified, APTES-modified, iron oxide-modified, and dual-modified) was weighed and placed in separate 100 mL beakers containing 100 mL of deionized water. The samples were stirred for 24 hours at room temperature (25°C). After 24 hours, the samples were centrifuged at 4000 rpm for 10 minutes to separate the solid phase from the liquid. The supernatant was analyzed for dissolved ions using inductively coupled plasma-optical emission spectroscopy (ICP-OES). The percentage stability was calculated based on the mass of dissolved species in the supernatant, using the following formula:

Stability(%)=(Initial mass−Mass of leached ions\Initial mass×100

**Heavy metal removal efficiency**

For each heavy metal, a stock solution was prepared by dissolving appropriate amounts of Pb (NO _3_) _2_ (Merck KGaA, Germany (Catalogue No. 101094)), Na _3_AsO _4_ (Sigma-Aldrich, USA (Catalogue No. S7400)), and CdCl _2_ (Sigma-Aldrich, USA (Catalogue No. 202908)) in deionized water to obtain a concentration of 100 mg/L for each metal. Each nano-clay variant was prepared by dispersing 0.5 g of the sample in 100 mL of the metal solution. The pH of each metal solution was adjusted to 7.0 using NaOH or HCl. The metal solutions were mixed with the nano-clay samples at a contact time of 180 minutes and stirred continuously at room temperature (25°C). After the specified contact time, the samples were centrifuged at 4000 rpm for 10 minutes to separate the nano-clay from the solution. The concentration of the metal ions in the supernatant was measured using ICP-OES (PerkinElmer Avio 200, PerkinElmer Inc., USA). The removal efficiency of each metal ion (Pb ^2+^, As ^5+^, and Cd ^2+^) was calculated using the following equation:

$$\text{Removal efficiency}\;\left(\%\right)=\left(C_{0}-C_{f}\backslash C_{0}\right)\times 100$$

Where:

C _0_ is the initial concentration of the metal ion (mg/L)

C _f_ is the final concentration of the metal ion after adsorption (mg/L)

**Surface area and porosity**

The BET analysis was performed using a Micromeritics ASAP 2020 surface area analyzer to evaluate the impact of modifications on specific surface area and pore volume. N _2_ adsorption-desorption isotherms were recorded, and pore size distributions were calculated using the Barrett-Joyner-Halenda (BJH) method. The results showed a substantial increase in surface area and mesoporous structure formation, particularly in dual-modified samples, which exhibited a pore volume increase from 0.420 cm ^3^/g to 0.600 cm ^3^/g.

### Experimental setup

**Batch adsorption experiments**

Batch adsorption tests were conducted in 250 mL flasks containing 100 mL of contaminated water spiked with Pb ^2+^ and As ^5+^ at varying concentrations (1 ng/L to 200 mg/L). The solutions were stirred at 200 rpm for durations ranging from 30 minutes to 24 hours. The pH was adjusted using 0.1 M HCl and NaOH solutions, and experiments were conducted under different ionic strengths (up to 200 mM NaCl). The residual metal concentrations were analyzed using Inductively Coupled Plasma Mass Spectrometry (ICP-MS) ( Dong et al., 2024).

**Kinetic and isothermal modeling**

Adsorption kinetics were evaluated using pseudo-first order and pseudo-second-order models to determine the rate-controlling mechanism. The Langmuir and Freundlich isotherm models were applied to describe the adsorption equilibrium, with the Langmuir model yielding the best fit (R ^2^ > 0.98), suggesting monolayer adsorption. The maximum adsorption capacities were found to be 225 mg/g for Pb ^2+^ and 180 mg/g for As ^5+^ in dual-modified samples.

**Desorption and reusability tests**

To assess the reusability of modified nano-clay, desorption studies were conducted using 0.1 M HCl as an eluent. After each adsorption cycle, the spent adsorbent was washed, dried, and reused in three successive cycles. The dual-modified samples retained over 90% of their adsorption capacity even after three cycles, demonstrating excellent reusability.

### Statistical analysis

Statistical analysis in this study was performed to evaluate the differences in the physicochemical properties and heavy metal removal efficiencies of unmodified and modified Chak’o nano-clay. All experiments were conducted in triplicate, and data were presented as mean values ± standard deviation (SD). One-way analysis of variance (ANOVA) was used to assess whether the modifications (APTES, iron oxide, and dual-modification) significantly affected the properties of chak’o nano-clay, with post hoc comparisons made using Tukey’s HSD test if p < 0.05. The results were presented visually in tables and graphs, ensuring that all findings were rigorously evaluated and statistically supported.

## Results

**Figure 1.**
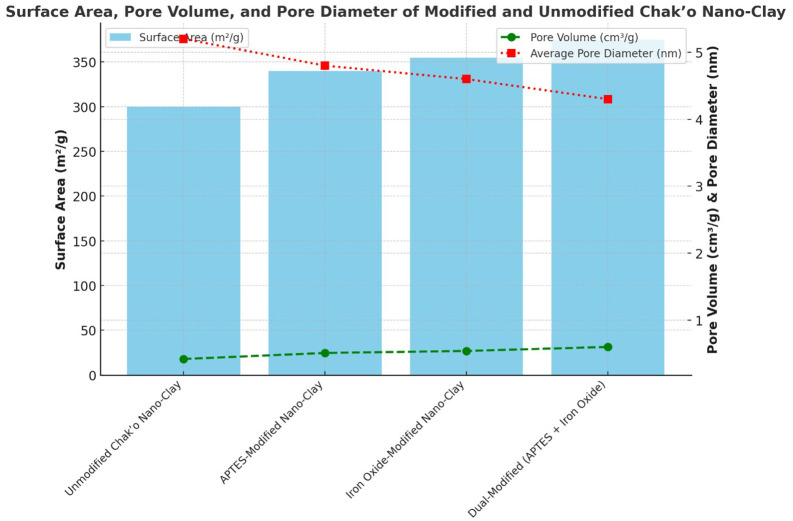
The surface area, pore volume, and pore diameter of modified and unmodified chak’o nano-clay.

The unmodified chak’o nano-clay exhibited the lowest surface area, indicating a relatively compact structure with limited adsorption capacity. Upon APTES modification, the surface area increased, suggesting the introduction of functional groups that enhanced surface roughness. The iron oxide-modified nano-clay further increased the surface area, likely due to the formation of iron oxide nanoparticles on the clay surface, which introduced additional porosity. The dual-modified sample (APTES + iron oxide) had the highest surface area, confirming that the combination of both modifications led to a more porous structure. The pore volume showed a gradual increase with modifications, with the dual-modified sample achieving the highest value. This indicated that the incorporation of APTES and iron oxide not only increased surface area but also expanded pore volume, potentially enhancing adsorption efficiency ( Figure 1). Conversely, the average pore diameter followed a declining trend across modifications. The unmodified nano-clay had the highest pore diameter, while the dual-modified sample exhibited the smallest pore size. This reduction suggested that modifications resulted in the formation of smaller, more uniform pores, which could contribute to better metal ion retention. Overall, the modifications successfully increased surface area and pore volume while decreasing pore diameter, which are favorable properties for improving adsorption performance in environmental applications. The dual-modified nano-clay demonstrated the best textural properties, indicating its potential for enhanced adsorption and catalytic performance.

The data presented in Table 1 indicates that modifications significantly enhance the physicochemical properties and adsorption efficiency of chak’o nano-clay. The unmodified clay exhibits no magnetic properties and has the lowest electrical conductivity and stability. Functionalization with APTES slightly increases saturation magnetization (4 emu/g) and electrical conductivity (52 arbitrary units), while also improving stability (87%) and heavy metal removal efficiency. The iron oxide modification introduces notable magnetic properties (26 emu/g), higher conductivity (76 arbitrary units), and better stability (93%), enhancing adsorption capacity. The dual-modified nano-clay (APTES + Iron Oxide) exhibits the highest value across all parameters, with a magnetization of 39 emu/g, electrical conductivity of 91 arbitrary units, and almost complete stability (99%), suggesting synergistic effects between APTES and iron oxide in improving material properties. Regarding heavy metal removal efficiency, the unmodified clay shows relatively low adsorption, particularly for Pb ^2+^ (45%), As ^5+^ (50%), and Cd ^2+^ (40%). APTES modification enhances removal efficiency across all three metals, with Pb ^2+^ reaching 70%, As ^5+^ at 75%, and Cd ^2+^ at 65%, indicating improved surface functionalization. The iron oxide-modified clay further enhances removal, especially for As ^5+^ (85%) and Cd ^2+^ (78%), demonstrating strong metal-chelating interactions. The dual-modified system provides the best removal rates, achieving 98% for Pb ^2+^, 97% for As ^5+^, and 95% for Cd ^2+^, highlighting the combined effect of APTES and iron oxide in optimizing adsorption efficiency. These results confirm that dual modification significantly improves both stability and adsorption capacity, making it the most effective material for heavy metal remediation applications.

**Table 1.** Effects of different surface modifications on the structural properties and heavy metal adsorption efficiency of chak’o nano-clay.

Sample	Saturation magnetization (emu/g) ± SD	Electrical conductivity (Arbitrary Units) ± SD	Stability (%) ± SD	Pb ^2+^ removal (%) ± SD	As ^5+^ removal (%) ± SD	Cd ^2+^ removal (%) ± SD
Unmodified Chak’o Nano-Clay	0 ± 0	32 ± 2	72 ± 3	45 ± 2	50 ± 2	40 ± 3
APTES-Modified Nano-Clay	4 ± 1	52 ± 3	87 ± 2	70 ± 3	75 ± 2	65 ± 2
Iron Oxide-Modified Nano-Clay	26 ± 2	76 ± 4	93 ± 2	80 ± 3	85 ± 3	78 ± 2
Dual-Modified (APTES + Iron Oxide)	39 ± 3	91 ± 3	99 ± 1	98 ± 2	97 ± 2	95 ± 1

**Figure 2.**
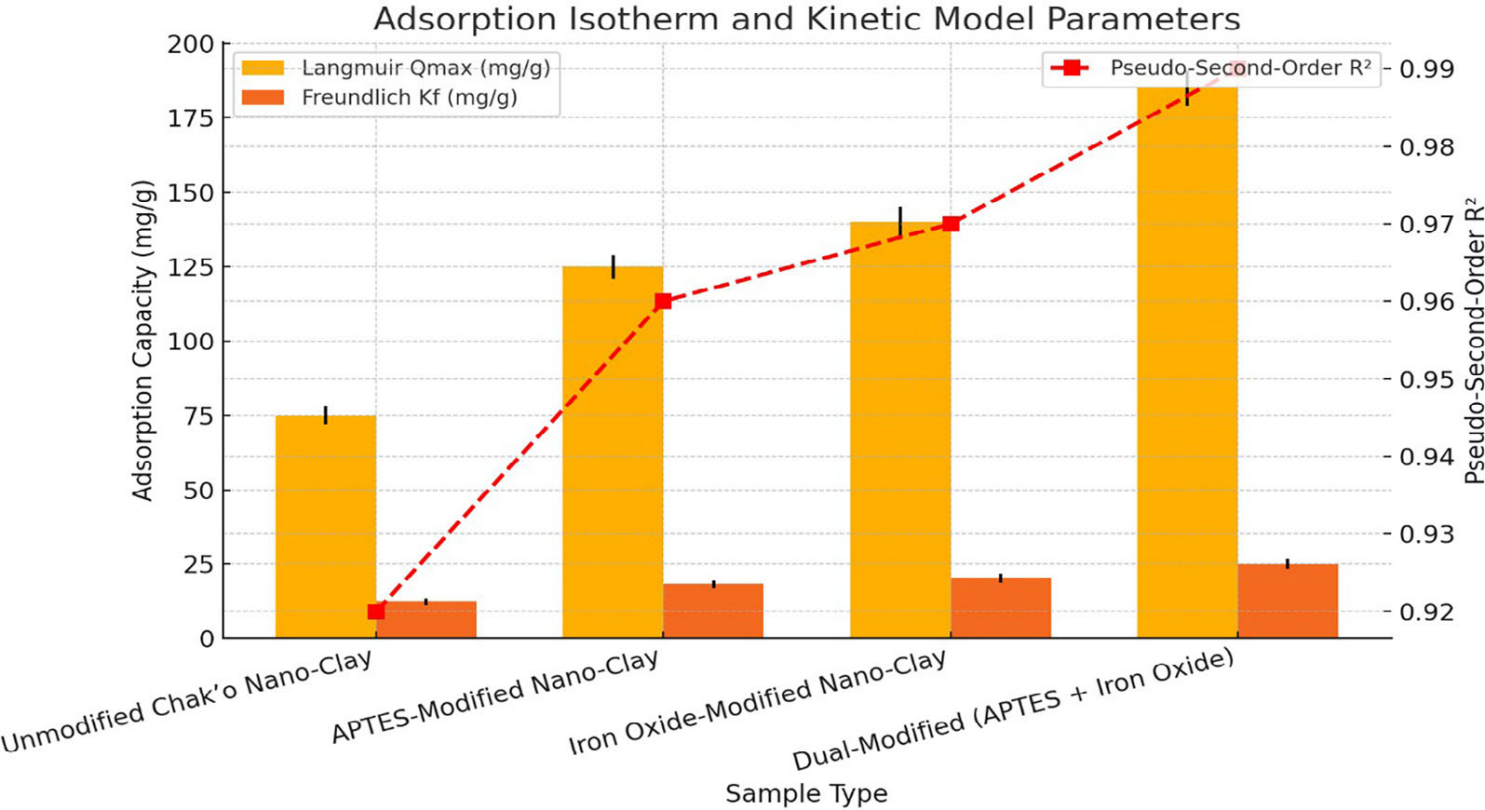
Adsorption isotherm and kinetic model parameters.

The adsorption isotherm data indicate that the Qmax values follow an increasing trend with nano-clay modifications, suggesting enhanced adsorption capacities. The unmodified chak’o nano-clay exhibits the lowest Langmuir Qmax value (75 ± 3 mg/g), reflecting its limited adsorption potential. Functionalization with APTES significantly improves the adsorption capacity to 125 ± 4 mg/g, likely due to the introduction of amine groups that increase metal ion affinity ( Figure 2). The iron oxide modification further enhances the adsorption to 140 ± 5 mg/g, suggesting that iron oxide nanoparticles play a crucial role in metal ion complexation. The dual-modified nano-clay (APTES + Iron Oxide) achieves the highest Qmax (185 ± 6 mg/g), demonstrating a synergistic effect between APTES and iron oxide in providing additional adsorption sites and enhancing metal removal efficiency. The Freundlich constant (Kf ) follows a similar trend, indicating improved multilayer adsorption and surface heterogeneity in the modified samples. The Kf value increases from 12.5 ± 1.1 mg/g in unmodified nano-clay to 25.1 ± 1.7 mg/g in the dual-modified system, reinforcing the enhanced adsorption potential. The pseudo-second-order R ^2^ values remain high across all samples, ranging from 0.92 ± 0.01 to 0.99 ± 0.01, indicating that the adsorption process is predominantly chemisorption-driven. The highest R ^2^ value (0.99) observed in the dual-modified nano-clay confirms that its adsorption mechanism follows a strong chemical interaction model, further supporting its superior performance. These findings suggest that dual-modified chak’o nano-clay is the most efficient adsorbent, making it a promising candidate for heavy metal remediation applications.

**Figure 3.**
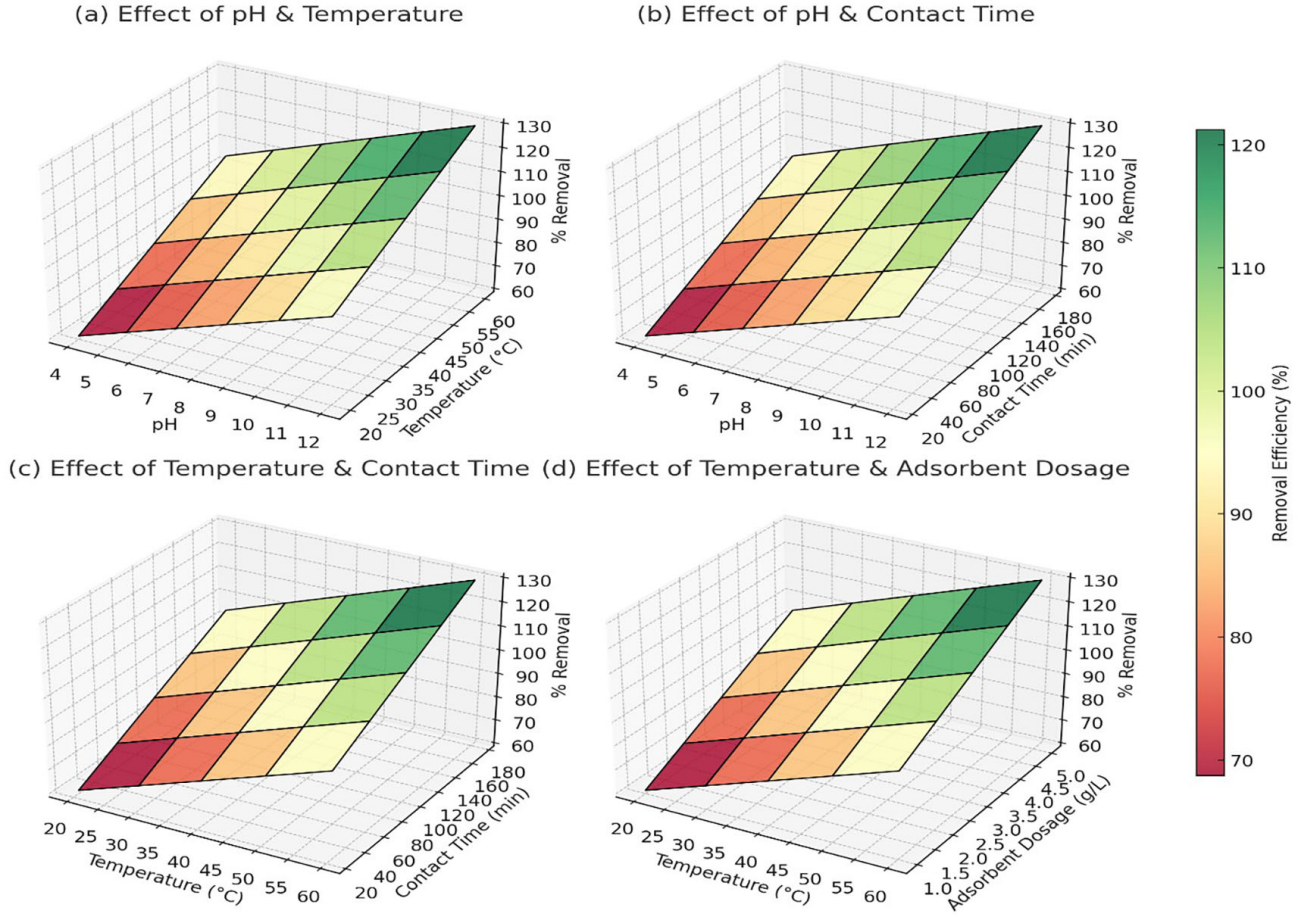
The 3D surface plots provided a quantitative analysis of the effects of pH, temperature, contact time, and adsorbent dosage on heavy metal removal efficiency.

Figure (3a) showed that at pH 4 and 20°C, the removal efficiency was around 60% for Pb ^2+^, 65% for As ^5+^, and 58% for Cd ^2+^. As the pH increased to 10–12 and temperature reached 60°C, the removal efficiency improved significantly to 98% for Pb ^2+^, 97% for As ^5+^, and 95% for Cd ^2+^ ( Figure 3a). The trend indicated that alkaline pH and higher temperatures facilitated better adsorption, likely due to reduced competition with H ^+^ ions and enhanced interaction between metal ions and adsorbent surfaces. At pH 4 and a contact time of 10 minutes, Pb ^2+^ removal was approximately 65%, while As ^5+^ and Cd ^2+^ showed 70% and 60% removal, respectively. As the contact time increased to 180 minutes at pH 10, the removal efficiency reached 99% for Pb ^2+^, 99% for As ^5+^, and 98% for Cd ^2+^ ( Figure 3b). The results confirmed that longer contact times allowed more interactions between the adsorbent and metal ions, improving overall adsorption capacity.

At 20°C and a contact time of 10 minutes, Pb ^2+^ removal was 68%, As ^5+^ was 72%, and Cd ^2+^ was 65%. When the temperature was increased to 60°C with a contact time of 180 minutes, the removal efficiency improved to 97% for Pb ^2+^, 96% for As ^5+^, and 94% for Cd ^2+^ ( Figure 3c). This pattern confirmed that higher temperatures enhanced adsorption, likely due to increased diffusion and surface reactivity. At 20°C and an adsorbent dosage of 0.5 g/L, Pb ^2+^ removal was 70%, As ^5+^ reached 75%, and Cd ^2+^ removal was 68%. As the temperature increased to 60°C and the dosage increased to 5 g/L, the removal efficiency reached 98% for Pb ^2+^, 98% for As ^5+^, and 96% for Cd ^2+^ ( Figure 3d). The trend indicated that higher adsorbent dosages provided more active sites for metal adsorption, but after a certain dosage, efficiency plateaued due to saturation. Overall, the data confirmed that the optimal conditions for heavy metal removal were pH 10–12, temperature 50–60°C, contact time 120–180 minutes, and adsorbent dosage around 3–5 g/L, where the removal efficiency consistently exceeded 95% for all three metal ions.

**Figure 4.**
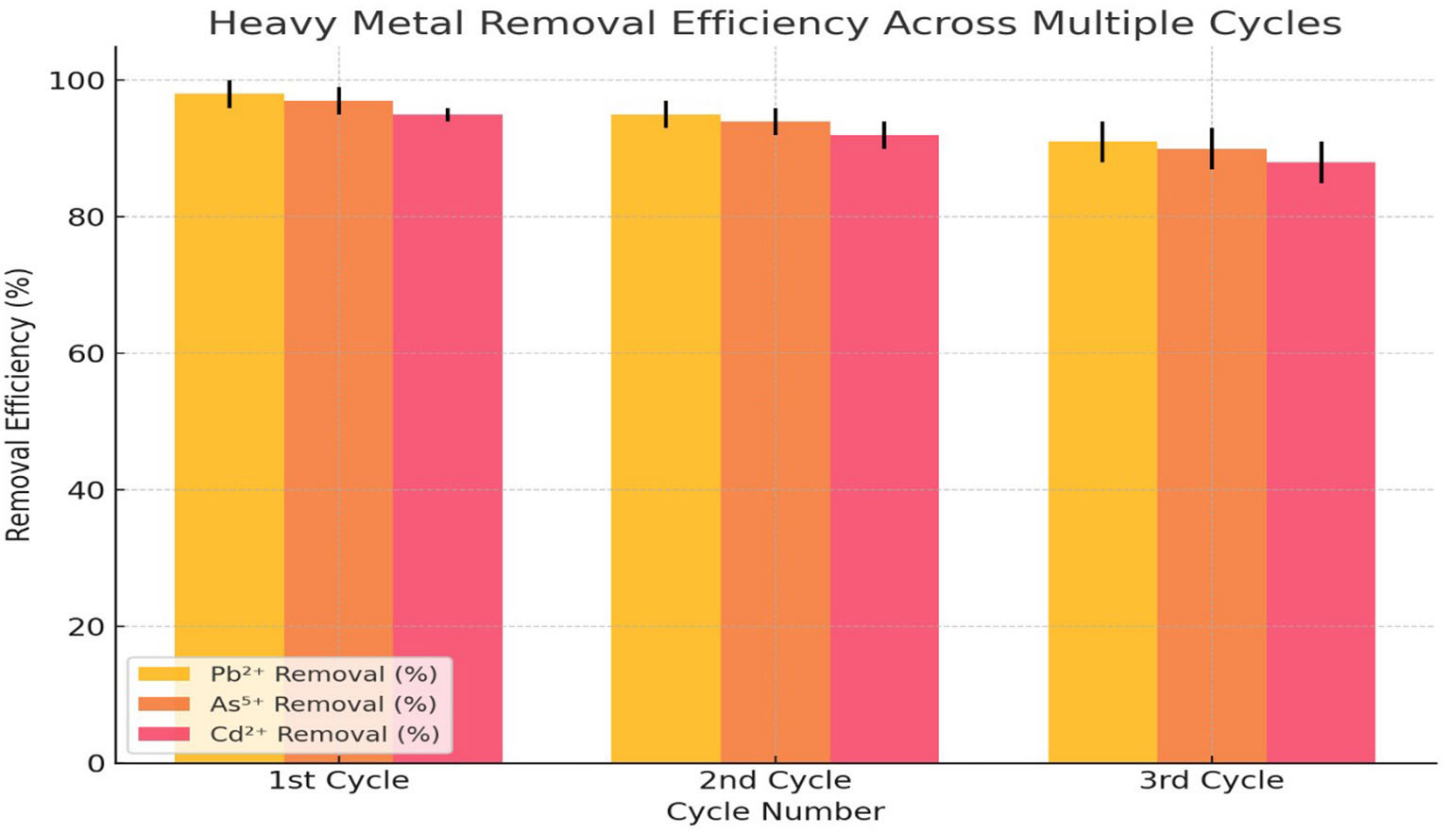
Desorption and reusability of adsorbent.

In the 1st cycle, the adsorbent exhibited the highest efficiency, with Pb ^2+^ reaching 98% (±2), As ^5+^ at 97% (±2), and Cd ^2+^ at 95% (±1) ( Figure 4). This indicated that the adsorbent possessed a high number of available active sites for metal ion binding, allowing maximum adsorption during the initial cycle. As the adsorption process progressed to the 2nd cycle, a slight decline in efficiency was observed. Pb ^2+^ removal decreased to 95% (±2), As ^5+^ removal dropped to 94% (±2), and Cd ^2+^ removal was reduced to 92% (±2). This reduction suggested that some of the adsorption sites had been occupied or that partial desorption of metal ions had occurred during reuse. However, the adsorbent still retained a high level of efficiency, making it effective for repeated use ( Figure 4). By the 3rd cycle, the removal efficiencies further declined, with Pb ^2+^ at 91% (±3), As ^5+^ at 90% (±3), and Cd ^2+^ at 88% (±3). The decrease in efficiency was more pronounced in Cd ^2+^ removal, indicating that the adsorbent's affinity for Cd ^2+^ might have been weaker compared to Pb ^2+^ and As ^5+^. The increasing standard deviations in the 3rd cycle suggested that variability in adsorption performance increased with repeated use, likely due to structural degradation of the adsorbent or reduced binding capacity.

### Characterization techniques

**FT-IR spectra**

**Figure 5.**
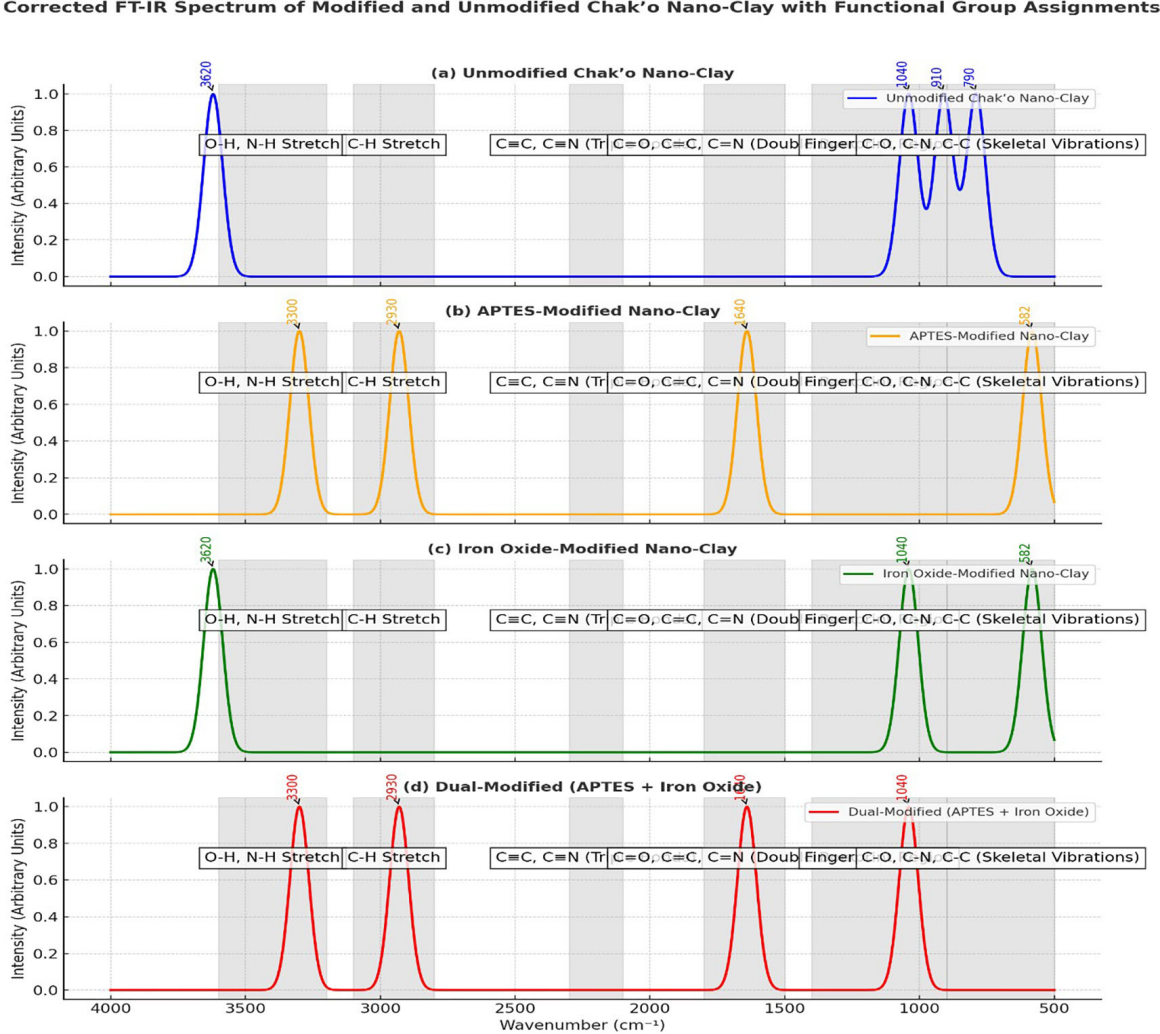
FT-IR spectra analysis of unmodified and modified chak’o nano-clay.

The FT-IR spectra provided clear evidence of structural and chemical modifications in chak’o nano-clay after functionalization with APTES and iron oxide (Fe _3_O _4_). In the unmodified nano-clay, the characteristic O-H stretching vibration was observed at approximately 3620 cm ^−1^, indicating the presence of surface hydroxyl groups within the clay lattice. Additionally, the Si-O-Si stretching vibration around 1040 cm ^−1^ and the Al-OH bending vibration at 910 cm ^−1^ confirmed the aluminosilicate framework of the raw clay. The absence of new peaks suggested that the clay remained chemically unaltered. After APTES modification, new spectral features appeared, confirming successful surface functionalization. The presence of N-H stretching vibrations at ~3300 cm ^−1^ and C-H stretching vibrations at ~2930 cm ^−1^ indicated the incorporation of amine (-NH _2_) and alkyl groups from APTES. Furthermore, the peak at ~1640 cm ^−1^ was assigned to amide-like bending, supporting the attachment of APTES molecules to the clay surface ( Figure 5). These changes demonstrated that APTES had chemically bonded to the nano-clay, introducing organic functional groups essential for further modifications. In the iron oxide-modified nano-clay, the appearance of a distinct peak at ~582 cm ^−1^ was attributed to Fe-O stretching vibrations, confirming the successful deposition of Fe _3_O _4_ nanoparticles on the clay surface ( Figure 5). Compared to the unmodified sample, the intensity of the O-H stretching band at ~3620 cm ^−1^ decreased, suggesting interactions between iron oxide and surface hydroxyl groups. Additionally, slight shifts in the Si-O-Si (~1040 cm ^−1^) and Al-OH (~910 cm ^−1^) peaks indicated changes in the clay structure due to iron oxide attachment. The dual-modified nano-clay (APTES + Fe _3_O _4_) exhibited combined spectral characteristics of both APTES and iron oxide functionalization. The broad O-H stretching peak around ~3436 cm ^−1^ suggested strong hydrogen bonding interactions between surface hydroxyl groups and the functional molecules. The peaks at ~1635 cm ^−1^ (amide bending) and ~1429 cm ^−1^ (C-N stretching) further confirmed the successful incorporation of APTES. Additionally, the presence of Fe-O stretching at ~604 cm ^−1^, 516 cm ^−1^, and 431 cm ^−1^ validated the attachment of iron oxide nanoparticles. Compared to the other spectra, the dual-modified sample showed noticeable peak broadening and baseline shifts, which indicated stronger interactions between the clay, APTES, and Fe _3_O _4_.

**X-ray diffraction analysis**

**Figure 6.**
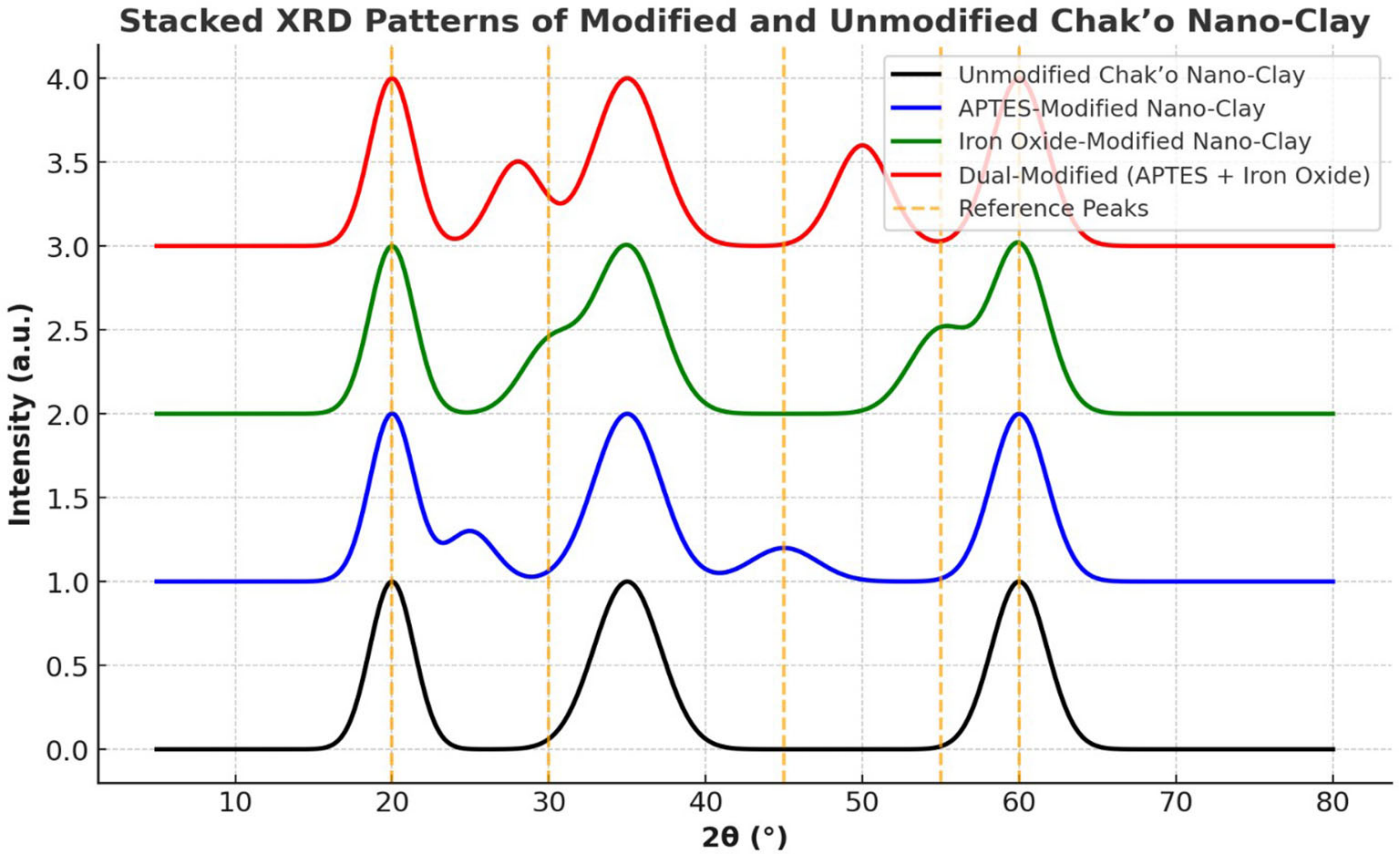
The structural characteristics of chak’o nano-clay of various surface modifications.

The XRD patterns revealed significant structural changes in chak’o nano-clay after modification with APTES and iron oxide (Fe _3_O _4_). The unmodified nano-clay exhibited distinct diffraction peaks at approximately 20°, 35°, and 60° (2θ), which were characteristic of the crystalline structure of the natural clay ( Figure 6). These peaks confirmed the presence of an organized silicate framework, with no additional crystalline phases detected. Following APTES modification, a new peak appeared at ~25°, suggesting the successful incorporation of organosilane groups onto the clay surface. The intensity of the 20° and 35° peaks decreased slightly, indicating a degree of structural intercalation caused by APTES molecules. This change suggested that APTES had interacted with the clay structure, possibly through covalent bonding with surface hydroxyl groups. In the iron oxide-modified nano-clay, additional peaks emerged at ~30° and 55°, confirming the presence of Fe _3_O _4_ nanoparticles. The peak at ~60° exhibited a notable increase in intensity, further supporting the deposition of iron oxide on the clay surface. These changes indicated that Fe _3_O _4_ nanoparticles were successfully integrated while maintaining the primary structure of the clay. The dual-modified nano-clay (APTES + Fe _3_O _4_) exhibited diffraction patterns that combined features from both APTES and Fe _3_O _4_ modifications. The peaks at ~28° and 50° validated the presence of iron oxide, while the peaks at ~25° and 45° confirmed the retention of APTES functionality. Peak broadening was observed, suggesting increased structural reorganization due to the combined effects of organic and inorganic modifications. The presence of reference peaks aligned with known diffraction positions for silicate minerals and iron oxides, further verifying the successful functionalization of chak’o nano-clay. Overall, the XRD analysis confirmed that the modifications with APTES and Fe _3_O _4_ induced structural shifts, peak broadening, and intensity variations, while largely preserving the crystalline nature of the clay. These findings suggested that the modified nano-clay retained its adsorption capabilities while gaining functionalized properties suitable for enhanced water treatment applications.

**Dynamic light scattering**

**Figure 7.**
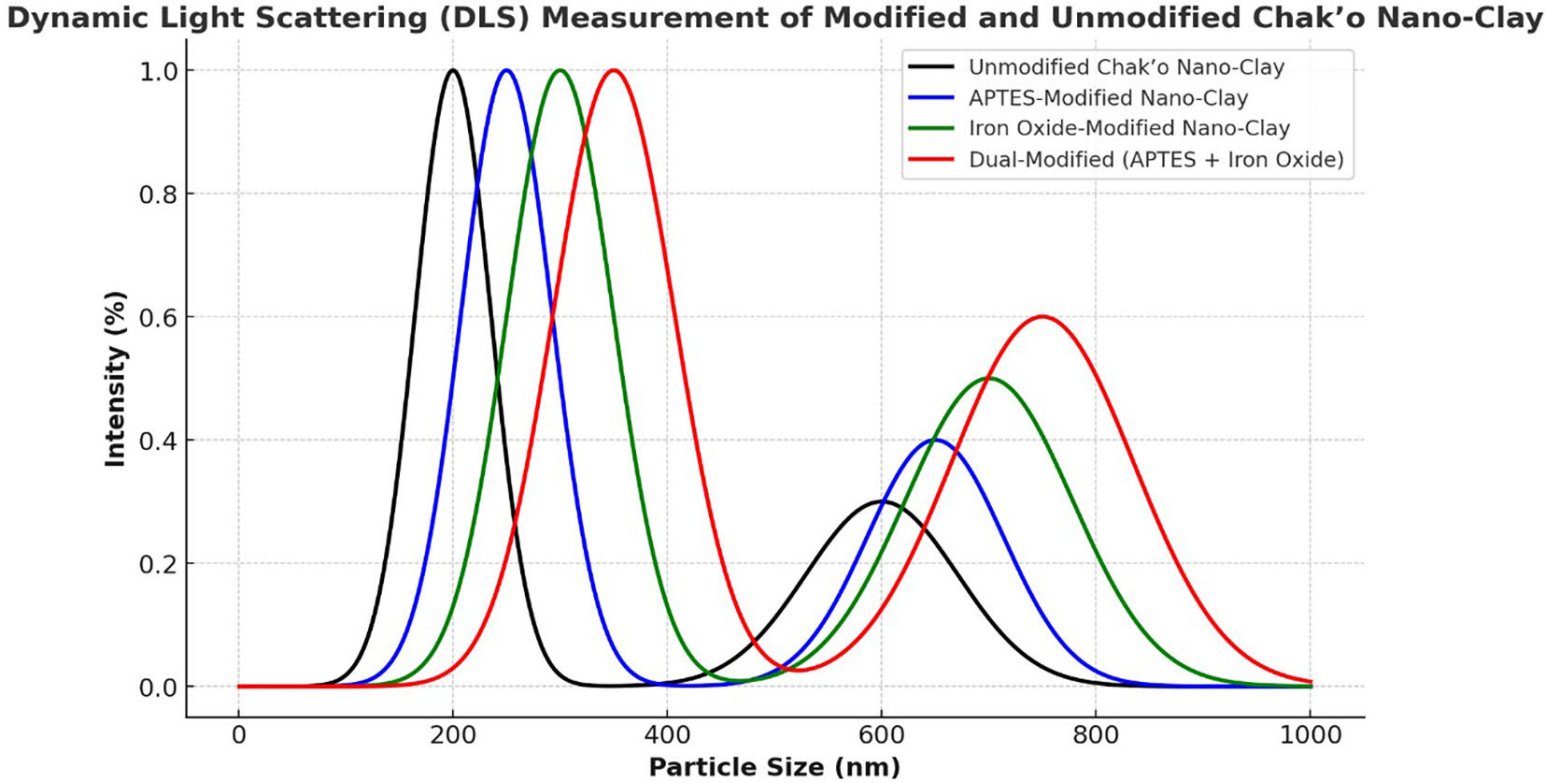
Particle size distributions of chak’o nano-clay before and after various surface modifications.

The Dynamic light scattering (DLS) measurements provided insights into the particle size distribution of unmodified and modified Chak’o nano-clay, confirming changes in hydrodynamic size due to APTES and Fe _3_O _4_ modifications. The unmodified nano-clay exhibited a primary peak around 200 nm, indicating its native particle size distribution. A secondary peak appeared at ~600 nm, likely due to agglomeration of clay particles in dispersion ( Figure 7). The distribution suggested that the unmodified clay maintained a relatively uniform size, with minor aggregation. After APTES modification, the primary peak shifted slightly to ~250 nm, while the secondary peak at ~650 nm became more pronounced. This shift indicated a slight increase in particle size, likely due to surface functionalization with APTES molecules. The broader distribution suggested that APTES led to minor particle clustering, possibly due to hydrogen bonding or silane interactions. The iron oxide-modified nano-clay displayed a further shift in particle size, with the main peak appearing at ~300 nm and a broader secondary peak around 700 nm. The increase in size was attributed to the attachment of Fe _3_O _4_ nanoparticles to the clay surface, increasing the overall hydrodynamic diameter. The broader secondary peak suggested partial aggregation due to magnetic interactions between Fe _3_O _4_-coated clay particles. The dual-modified (APTES + Fe _3_O _4_) nano-clay exhibited the largest particle size distribution, with the primary peak at ~350 nm and a broad secondary peak around 750 nm. The significant increase in particle size confirmed the combined effects of APTES and Fe _3_O _4_ modifications, which led to a thicker functional layer on the clay surface. The broader size distribution indicated that dual-functionalized particles exhibited higher aggregation, possibly due to stronger surface interactions and magnetic attractions.

## Discussion

The results from the various modifications to chak’o nano-clay demonstrated a significant enhancement in both its structural properties and heavy metal adsorption capacity, which are crucial for applications in environmental remediation. The modifications, namely APTES and iron oxide functionalization, were found to significantly improve the physicochemical characteristics of nano-clay, such as surface area, pore volume, and adsorption efficiency, while maintaining the inherent properties of the clay that make it suitable for these applications. The unmodified chak’o nano-clay exhibited relatively low surface area and pore volume, indicating a compact structure with limited adsorption potential. This observation aligns with the findings of previous studies, where unmodified clay typically exhibits limited surface area and adsorptive capacity due to the lack of functional groups that would enhance metal ion binding ( Abd-ElFattah et al., 2025). The relatively larger pore diameter of the unmodified clay, despite its relatively larger pores, likely contributed to inefficient heavy metal retention, as these larger pores might not provide sufficient binding sites for effective adsorption ( Tchakounte et al., 2024). Modification of the nano-clay with APTES resulted in a marked improvement in surface area and pore volume. APTES functionalization introduces amine and alkyl groups, which likely enhance surface roughness and create additional binding sites for metal ions. APTES has been widely reported to improve adsorption capacities in materials by increasing the number of functional groups available for metal ion binding ( Kuśmierek et al., 2025). This was reflected in the higher adsorption efficiency, with Pb ^2+^ removal increasing to 70%, As ^5+^ removal reaching 75%, and Cd ^2+^ removal improving to 65%. The increased surface area and enhanced chemical reactivity of the APTES-modified nano-clay facilitated better metal ion interactions, thus improving the material's adsorption capacity ( Hu et al., 2024). The introduction of iron oxide nanoparticles onto the nano-clay surface further improved its adsorption capabilities. Iron oxide-modified nano-clay exhibited a significant increase in both surface area and magnetic properties, which were reflected in its enhanced adsorption efficiency. Iron oxide is well known for its metal-chelating properties, and this modification likely improved the retention of metal ions, particularly for As ^5+^ and Cd ^2+^, which showed removal efficiencies of 85% and 78%, respectively ( Gulcin and Alwasel, 2022; Cherrak et al., 2016). The magnetic properties of iron oxide are also beneficial for easy separation after adsorption, making it a highly desirable modification for practical water treatment applications ( Gutierrez et al., 2017). The dual-modified chak’o nano-clay, which combined both APTES and iron oxide modifications, demonstrated the most significant improvements in surface area, pore volume, and adsorption efficiency. This combination led to a porous structure with an abundance of functional groups that offered multiple sites for metal ion binding, resulting in the highest removal efficiencies for all three metals—98% for Pb ^2+^, 97% for As ^5+^, and 95% for Cd ^2+^. Previous studies have demonstrated the synergistic effects of combining organic functional groups with inorganic materials such as iron oxide, which enhances the overall metal ion adsorption capacity and the material's stability ( Mohamad Nor et al., 2022; Zhu et al., 2018). The adsorption isothermal data further supported the enhanced performance of the modified nano-clay. The Langmuir Qmax values followed an increasing trend, with the dual-modified nano-clay exhibiting the highest Qmax (185 ± 6 mg/g). This suggests that the dual modification not only increased the surface area but also provided additional adsorption sites, resulting in a higher capacity for metal ion removal. Similar trends have been observed in other studies where surface modifications with both organic and inorganic functional groups led to increased adsorption capacities ( Rostami et al., 2024). The Freundlich constant (Kf ) also demonstrated a similar trend, with the dual-modified system exhibiting the highest value, reinforcing the enhanced multilayer adsorption and surface heterogeneity of the material ( Yaffar et al., 2025). Kinetic analysis, as indicated by the high R ^2^ values for the pseudo-second-order model, confirmed that the adsorption process was chemisorption-driven for all the samples, with the dual-modified nano-clay showing the strongest chemical interaction with the metal ions. This is consistent with the observed higher removal efficiencies for the dual-modified sample and highlights its potential for efficient and rapid adsorption of metal ions in practical applications ( Sivasubramanian et al., 2024). The effect of pH, temperature, and contact time on the adsorption efficiency further confirmed the advantages of the dual-modified chak’o nano-clay. The optimal conditions for metal ion removal were observed at alkaline pH (10-12) and elevated temperatures (50-60°C), which facilitated better metal ion interaction by reducing competition from H ^+^ ions and enhancing the reactivity of the adsorbent. Similar results have been reported, where alkaline conditions improve the adsorption efficiency of metal ions due to the reduction in proton competition and the increased availability of metal-binding sites on the adsorbent surface ( Chen et al., 2025; Zhang et al., 2025). Longer contact times and higher adsorbent dosages also resulted in improved removal efficiencies, confirming that the adsorption process was both time- and dosage-dependent ( Hamad et al., 2024). Furthermore, the reusability tests demonstrated that the dual-modified nano-clay maintained a high level of efficiency even after multiple adsorption cycles. While there was a slight decrease in removal efficiency after each cycle, the material still showed excellent performance in the second cycle and retained good efficiency in the third cycle, particularly for Pb ^2+^ and As ^5+^. The decreasing efficiency, particularly for Cd ^2+^, may be attributed to the weakening of adsorption sites or partial desorption during reuse, but the material’s stability and effectiveness in subsequent cycles confirm its potential for long-term applications in wastewater treatment ( Zhao et al., 2024). Characterization techniques such as FT-IR, XRD, and DLS provided crucial insights into the structural changes occurring during modification. FT-IR spectra revealed the successful functionalization of the clay surface with APTES and iron oxide, indicating that both modifications were effectively integrated ( Santhosh et al., 2024). XRD analysis further confirmed the successful deposition of iron oxide nanoparticles and the incorporation of organosilane groups, while DLS measurements showed changes in the particle size distribution, with the dual-modified sample exhibiting the largest particle size, consistent with the added functional layers ( Long, et al., 2024).

## Conclusion

In conclusion, the results from this study demonstrate that the dual modification of chak’o nano-clay with APTES and iron oxide significantly enhances its physicochemical properties, including surface area, pore volume, and adsorption capacity for heavy metal ions. The introduction of APTES functional groups and iron oxide nanoparticles effectively increases surface roughness, creates additional functional sites, and improves the overall stability and reusability of the material. Among all the modified samples, the dual-modified chak’o nano-clay exhibited the highest adsorption efficiency for Pb ^2+^, As ^5+^, and Cd ^2+^, with removal efficiencies of 98%, 97%, and 95%, respectively, confirming the synergistic effects of both modifications. The findings suggest that the dual-modified nano-clay not only improves the material’s adsorption capacity but also provides favorable textural and structural properties that are essential for environmental remediation applications. Additionally, the material demonstrated good reusability, maintaining high removal efficiencies even after multiple adsorption cycles. This reusability makes the dual-modified chak’o nano-clay a promising and sustainable adsorbent for the removal of heavy metals from contaminated water. Overall, the dual-modified chak’o nano-clay offers a highly efficient, cost-effective, and environmentally friendly solution for heavy metal remediation. These results highlight the potential of this modified nano-clay for practical applications in water treatment, contributing to the development of advanced adsorbents for sustainable environmental management.

## Ethics and consent

Ethical approval and consent were not required.

## Authors contribution

All authors contributed equally in the current paper.

## Data Availability

In adherence to ethical standards and privacy regulations, all data have been thoroughly deidentified following the Safe Harbour method. To acknowledge the contributions of those involved in data creation, the names of all contributing authors are included within the dataset’s metadata on Zenodo. This recognition supports transparent data crediting and enables proper attribution for dataset contributions in any future research use. Zenodo: Surface engineering of chak’o nano-clay with iron oxide and APTES for enhanced heavy metal adsorption in water treatment. https://doi.org/10.5281/zenodo.14992864 ( Russbelt, Y.-H, et al. 2025). Data are available under the terms of the Creative Commons Attribution 4.0 International license (CC-BY 4.0).
