# Supplementary material for: Surface engineering of chak’o nano-clay with iron oxide and APTES for enhanced heavy metal adsorption in water treatment
Source: F1000Res. 2026 Jan 21;14:334. Originally published 2025 Mar 27. [Version 4] doi: 10.12688/f1000research.162878.4 (PMC12756587; doi:10.12688/f1000research.162878.4)
Supplement: Peer review history

# Reviewer report: Reviewer response for version 4

**DOI:** 10.5256/f1000research.195350.r452052

**Published:** 2026-02-04

**Authors:** Beatriz Miguel Hernandez

**Affiliations:** 1 Universidad Politecnica de Cartagena, Cartagena, Region of Murcia, Spain

The authors have revised the article and included the reviewers' considerations and clarifications. Therefore, I consider the article suitable for indexing.

Is the work clearly and accurately presented and does it cite the current literature?

Partly

If applicable, is the statistical analysis and its interpretation appropriate?

Partly

Are all the source data underlying the results available to ensure full reproducibility?

Partly

Is the study design appropriate and is the work technically sound?

Partly

Are the conclusions drawn adequately supported by the results?

Partly

Are sufficient details of methods and analysis provided to allow replication by others?

Yes

Reviewer Expertise:

NA

I confirm that I have read this submission and believe that I have an appropriate level of expertise to confirm that it is of an acceptable scientific standard.

# Reviewer report: Reviewer response for version 3

**DOI:** 10.5256/f1000research.192746.r444495

**Published:** 2025-12-31

**Authors:** Shuping Wu

**Affiliations:** 1 Jiangsu University, Zhenjiang, China

The authors have made the necessary modifications to their work, and the revised manuscript is considered to be accepted for indexing now.

Is the work clearly and accurately presented and does it cite the current literature?

Yes

If applicable, is the statistical analysis and its interpretation appropriate?

Partly

Are all the source data underlying the results available to ensure full reproducibility?

Partly

Is the study design appropriate and is the work technically sound?

Yes

Are the conclusions drawn adequately supported by the results?

Partly

Are sufficient details of methods and analysis provided to allow replication by others?

Yes

Reviewer Expertise:

Material science

I confirm that I have read this submission and believe that I have an appropriate level of expertise to confirm that it is of an acceptable scientific standard.

# Reviewer report: Reviewer response for version 2

**DOI:** 10.5256/f1000research.188301.r423209

**Published:** 2025-10-30

**Authors:** Beatriz Miguel Hernandez

**Affiliations:** 1 Universidad Politecnica de Cartagena, Cartagena, Region of Murcia, Spain

Review: Surface engineering of chak’o nano-clay with iron oxide and APTES for enhanced heavy metal adsorption in water treatment 

  

- Section “Binary (Pb-As) competitive adsorption. 

A T is missing at the beginning of the paragraph, and symbols have been included in the fourth line that may lead to confusion. 

  

- Section “Kinetic and isothermal modeling” 

The second part of this paragraph corresponds to the results of the isothermal fittings, as this part is due to the description of the methodology, the results of the Langmuir model should be moved to the results section. 

- Figure 1, The legend should not cover the surface area results. 
- Pag.8 first paragraph “...while decreasing pore diameter, which are favorable properties improving adsorption performance in environmental application” This is true when ionic metals are removed, but in some cases, organic compounds are the target of separation, in which case a larger pore diameter is required. 
- The authors state that the Langmuir model is the best fit, suggesting monolayer adsorption. However, in Figure 2, they include the adsorption capacity of the Freundlich fit and also calculate Kf from this model in paragraph 2, page 9 (without giving the R² values of the fit). Since the authors ruled out the Freundlich isotherm, what is the point of giving some of the results obtained in the fit? Incidentally, the R² values included in Figure 2 correspond to the pseudo-second-order fit, and not to the Langmuir fit, whose Q_max values are also represented, which is a little confusing. 

 

- Was the solution to the problem prepared in the laboratory? The authors are studying the adsorption of Pb ^+2^, Cd ^+2^, and As ^+5^ in aqueous solution by varying the pH. However, the ions occur in different chemical compounds depending on the pH. The arsenic (V) ion in aqueous solutions is typically found in the form of arsenate AsO₄³⁻, or its protonated forms, such as HAsO₄²⁻ and H₂AsO₄⁻, and the specific form present depends on the pH of the solution. As the pH increases to alkaline levels, Pb ^2+^ begins to form other species, such as the Pb(OH) ^+^ and Pb(OH)₂ ions.  Cd ^2+^ exists as a free ion in acidic to neutral solutions, but can form cadmium hydroxide species such as Cd(OH) ^+^ and Cd(OH) _2_ under more alkaline conditions. That is, depending on the pH of the solution, the authors are studying the adsorption of different species. 

Is the work clearly and accurately presented and does it cite the current literature?

Partly

If applicable, is the statistical analysis and its interpretation appropriate?

Partly

Are all the source data underlying the results available to ensure full reproducibility?

Partly

Is the study design appropriate and is the work technically sound?

Partly

Are the conclusions drawn adequately supported by the results?

Partly

Are sufficient details of methods and analysis provided to allow replication by others?

Yes

Reviewer Expertise:

chemical engeniering, adsoption, computational chemistry

I confirm that I have read this submission and believe that I have an appropriate level of expertise to state that I do not consider it to be of an acceptable scientific standard, for reasons outlined above.

# Response

**Published:** 2026-01-15

**Authors:** RUSSBELT YAULILAHUA HUACHO

**Affiliations:** Huancavelica, Universidad Nacional de Huancavelica, Huancavelica, Huancavelica, Peru

**Comment 1:** Section “Binary (Pb-As) competitive adsorption. A T is missing at the beginning of the paragraph, and symbols have been included in the fourth line that may lead to confusion. 

**Answer 1:** The paragraph in the “Binary (Pb–As) competitive adsorption” section has been revised. The missing initial “T” was corrected by rewriting the opening sentence. Additionally, all symbols were standardized and explicitly defined (q_e, C_e, K_d, and α_{Pb/As}) with consistent subscripts and units to remove ambiguity and prevent confusion.

**Comment 2:** Section “Kinetic and isothermal modeling” The second part of this paragraph corresponds to the results of the isothermal fittings, as this part is due to the description of the methodology, the results of the Langmuir model should be moved to the results section. 

**Answer 2:** We thank the reviewer for this observation. The “Kinetic and isothermal modeling” section has been revised to contain only methodological descriptions. All Langmuir fitting results and related interpretations have been moved to the Results section under adsorption isotherm analysis. This revision clearly separates methodology from results and improves the logical structure of the manuscript.

**Comment 3:** Figure 1, The legend should not cover the surface area results. 

**Answer 3:** New corrected figures have been made and added.

**Comment 4:** Pag.8 first paragraph “...while decreasing pore diameter, which are favorable properties improving adsorption performance in environmental application” This is true when ionic metals are removed, but in some cases, organic compounds are the target of separation, in which case a larger pore diameter is required. 

**Answer 4:** The statement has been revised to clarify that reduced pore diameter is advantageous for ionic metal adsorption, while acknowledging that larger pore sizes may be necessary for the removal of bulky organic contaminants. This modification avoids overgeneralization and improves conceptual accuracy.

**Comment 5:** The authors state that the Langmuir model is the best fit, suggesting monolayer adsorption. However, in Figure 2, they include the adsorption capacity of the Freundlich fit and also calculate Kf from this model in paragraph 2, page 9 (without giving the R² values of the fit). Since the authors ruled out the Freundlich isotherm, what is the point of giving some of the results obtained in the fit? Incidentally, the R² values included in Figure 2 correspond to the pseudo-second-order fit, and not to the Langmuir fit, whose Q_max values are also represented, which is a little confusing. 

**Answer 5:** We thank the reviewer for identifying this important inconsistency. The manuscript has been revised to remove Freundlich isotherm parameters from both the text and Figure 2, as the Freundlich model showed weaker correlations and was not retained for interpretation. Langmuir fitting results are now reported exclusively, with corresponding R² values explicitly provided. In addition, Figure 2 has been revised to include only Langmuir isotherm parameters, while kinetic (pseudo-second-order) R² values are presented separately to avoid mixing equilibrium and kinetic indicators. These changes eliminate ambiguity and ensure consistency between model selection, reported parameters, and graphical presentation.

**Comment 6:** Was the solution to the problem prepared in the laboratory? The authors are studying the adsorption of Pb ^+2^, Cd ^+2^, and As ^+5^ in aqueous solution by varying the pH. However, the ions occur in different chemical compounds depending on the pH. The arsenic (V) ion in aqueous solutions is typically found in the form of arsenate AsO₄³⁻, or its protonated forms, such as HAsO₄²⁻ and H₂AsO₄⁻, and the specific form present depends on the pH of the solution. As the pH increases to alkaline levels, Pb ^2+^ begins to form other species, such as the Pb(OH) ^+^ and Pb(OH)₂ ions.  Cd ^2+^ exists as a free ion in acidic to neutral solutions but can form cadmium hydroxide species such as Cd(OH) ^+^ and Cd(OH) _2_ under more alkaline conditions. That is, depending on the pH of the solution, the authors are studying the adsorption of different species. 

**Answer 6:** We thank the reviewer for this important observation. The manuscript has been revised to explicitly state that all Pb, Cd, and As(V) solutions were prepared in the laboratory using analytical-grade salts. We now clearly acknowledge that metal speciation varies with pH and that adsorption experiments therefore involve different dominant aqueous species depending on solution conditions. This clarification has been added to the Methods and further discussed in the Discussion section. The adsorption results are thus interpreted as interactions between the modified nano-clay and the prevailing metal or oxyanion species at each pH, which reflects environmentally relevant conditions rather than an idealized single-ion system.

# Reviewer report: Reviewer response for version 2

**DOI:** 10.5256/f1000research.188301.r423205

**Published:** 2025-10-30

**Authors:** Shuping Wu

**Affiliations:** 1 Jiangsu University, Zhenjiang, China

1.  The figure of N₂ adsorption-desorption isotherms should be provided.

 2.  How about the micromorphologies of the nano-clay, the corresponding SEM images are suggested to be offered.

 3. Figure 1 should be formatted as clear one, and the error bars should be added.

 4. Figure 2 is rough. 

 5. The captions in Figure 6 overlap the line, making sure them separate.

 6. Strengthen the mechanistic discussion linking surface chemistry to adsorption behavior in the section of Discussion.

 7. Elaborate on the physicochemical reason Pb²⁺ > As⁵⁺ > Cd²⁺

Is the work clearly and accurately presented and does it cite the current literature?

Yes

If applicable, is the statistical analysis and its interpretation appropriate?

Partly

Are all the source data underlying the results available to ensure full reproducibility?

Partly

Is the study design appropriate and is the work technically sound?

Yes

Are the conclusions drawn adequately supported by the results?

Partly

Are sufficient details of methods and analysis provided to allow replication by others?

Yes

Reviewer Expertise:

Material science

I confirm that I have read this submission and believe that I have an appropriate level of expertise to confirm that it is of an acceptable scientific standard, however I have significant reservations, as outlined above.

# Reviewer report: Reviewer response for version 1

**DOI:** 10.5256/f1000research.179148.r391942

**Published:** 2025-06-28

**Authors:** Thabang H Mokhothu

**Affiliations:** 1 School of Chemical and Physical Science, Univesity of Mpumalanga, Mpumalanga, South Africa

The paper addresses the fabrication of unmodified, APTES-modified, iron oxide-modified, and dual-modified chak’o nano-clay composites for the adsorption of heavy metal ions (lead (Pb ^2+^) and arsenic (As ^5+^)). Addressing environmental challenges stemming from heavy metal pollution is crucial in mitigating health risks associated with heavy metal pollution. The study addresses a relevant scope of the journal, presenting a novel approach to heavy metal ion removal from water bodies using modified chak’o nano-clay composites. Here are a couple of comments to the authors.

 Comments

 Characterisation techniques Section

 Under this section, the authors discussed the dynamic light scattering (DLS) results very well; however, the experimental procedure for the method followed for this analysis is not provided in the modification section.

 Results Section

 The high adsorption efficiency of the dual-modified Chak’o nano-clay composite for both metals is noteworthy. However, it would be valuable to know whether the authors examined the selectivity between the two metals. Additionally, assessing whether the composite can maintain similar adsorption efficiency in the simultaneous presence of both metals—simulating real water conditions—would further strengthen the study.

Is the work clearly and accurately presented and does it cite the current literature?

Yes

If applicable, is the statistical analysis and its interpretation appropriate?

Yes

Are all the source data underlying the results available to ensure full reproducibility?

Yes

Is the study design appropriate and is the work technically sound?

Yes

Are the conclusions drawn adequately supported by the results?

Yes

Are sufficient details of methods and analysis provided to allow replication by others?

Yes

Reviewer Expertise:

Polymers and composites, water pollution, and  nanomaterials.

I confirm that I have read this submission and believe that I have an appropriate level of expertise to confirm that it is of an acceptable scientific standard.

# Response

**Published:** 2025-09-16

**Authors:** RUSSBELT YAULILAHUA HUACHO

**Affiliations:** Huancavelica, Universidad Nacional de Huancavelica, Huancavelica, Huancavelica, Peru

Comment 1 — *DLS method not described in Methods*

**Answer:** Added a DLS experimental procedure in Methods → “Characterization techniques”.

 Comment 2 — *Selectivity between Pb²⁺ and As⁵⁺ not examined; performance in simultaneous presence not assessed*

**Answer:** We address selectivity in two complementary ways: (i) quantify **apparent selectivity** from single-solute isotherms (no new experiments needed) and (ii) add a **binary-system protocol** for future/ongoing tests.
